# Correlation between adenoma detection rate and other quality indicators, and its variability depending on factors such as sedation or indication for colonoscopy

**DOI:** 10.3389/fphar.2022.1041915

**Published:** 2022-12-19

**Authors:** Andrei Lucian Groza, Bogdan Silviu Ungureanu, Cristian Tefas, Bogdan Miuțescu, Marcel Tanțău

**Affiliations:** ^1^ Iuliu Hațieganu University of Medicine and Pharmacy, 3rd Department of Internal Medicine, Cluj-Napoca, Romania; ^2^ Research Center of Gastroenterology and Hepatology, University of Medicine and Pharmacy of Craiova, Craiova, Romania; ^3^ Department of Gastroenterology, University of Medicine and Pharmacy of Craiova, Craiova, Romania; ^4^ Regional Institute of Gastroenterology and Hepatology “Prof. dr. Octavian Fodor”, Cluj-Napoca, Romania; ^5^ Department of Gastroenterology and Hepatology, “Victor Babeș" University of Medicine and Pharmacy, Timisoara, Romania

**Keywords:** adenoma detection rate, screening colonoscopy, quality indicators, adenoma per colonoscopy, colorectal cancer, sedation

## Abstract

Colorectal cancer (CRC) is an important worldwide public health burden and colonoscopy is the main diagnostic and most importantly, preventive method. For this reason, many countries have implemented national or regional CRC screening programs. High-quality colonoscopy is a prerequisite to effectively detect premalignant lesions, like adenomas. The quality of colonoscopy is assessed using several quality indicators, the main one being adenoma detection rate (ADR). In Romania, despite CRC having the highest incidence of all cancers, there is no national screening program and quality in colonoscopy is not routinely assessed. We therefore wanted to evaluate the actual level of quality in colonoscopy in a region of Romania. Our study was conducted in two private endoscopy clinics over a period of 7 months. 1,440 consecutive colonoscopies performed by five physicians were included in the study. We found that the quality level is above the minimum one recommended by international societies and that the ADR calculation method does not significantly influence its value. Furthermore, ADR correlated well with other quality indicators such as polyp detection rate (PDR) and adenoma per colonoscopy (APC). An interesting finding was that ADR was higher among colonoscopies performed without sedation. Thus, our data encourage endoscopists to adopt a sedation-free colonoscopy in their practice without an impact on the quality of the procedure.

## Introduction

Colorectal cancer (CRC) is one of the worldwide leading causes of cancer death and colonoscopy is the most important method of screening and diagnosis. To provide accurate results, endoscopists must be sure that they have performed a high-quality colonoscopy. The quality of colonoscopy is routinely assessed in countries where there are national CRC screening programs, using several quality indicators. The main one is adenoma detection rate (ADR), representing the proportion of screening colonoscopies in which at least one adenoma has been detected. However, there is uncertainty in the literature regarding the inclusion or exclusion criteria of patients whose ADR is reported. The American Society of Gastrointestinal Endoscopy (ASGE) defines ADR as the proportion of screening colonoscopies in average-risk individuals aged 50 years or older in which at least one adenoma has been detected (Rizk et al., 2015). The minimum standard proposed by ASGE is 30% for male and 20% for female patients. The European Society of Gastrointestinal Endoscopy (ESGE) reports the ADR to the total number of screening and diagnostic colonoscopies in individuals aged 50 years or older, mentioning some exclusion criteria like workup of previously detected lesion or follow-up in inflammatory bowel disease (Kaminski et al., 2017). The minimum standard proposed by ESGE is 25% regardless of gender.

In Romania, despite CRC having the highest incidence of all cancers, there is no national screening program, thus screening is conducted opportunistically. In addition, quality in colonoscopy is not routinely assessed and there are no national guidelines for quality assessment**.**

In this study we wanted to determine the proportion of screening colonoscopies and the current level of quality in endoscopy in a region in Romania, based on the quality indicators recommended by ESGE. We also wanted to establish if there are any statistical differences in calculating ADR depending on the indication for colonoscopy, and to compare it with other newly proposed quality indicators like adenoma per colonoscopy (APC) and adenoma per positive participant (APP).

## Materials and methods

We conducted a prospective observational study in two private endoscopy clinics from two cities in northwestern Romania (Cluj-Napoca and Zalau), between July 1 and 31 December 2021.

We recorded all colonoscopies performed by five endoscopists with at least 5 years of experience and a minimum of 300 colonoscopies per year. The exclusion criteria were emergency colonoscopies, patients without a clear indication for colonoscopy, patients with indication for sigmoidoscopy or patients with a specific therapeutic indication. All endoscopies were performed using HD equipment with virtual chromoendoscopy and magnification. In both clinics the data were recorded electronically in a different system than the main patient record system, due to the lack of a standardized endoscopy reporting system.

The recorded data were related to patients’ demographics and quality of the procedures. The quality indicators we followed were time slot for colonoscopy, reason for admission, quality of bowel preparation (defined as adequate or inadequate, based on the Boston Bowel Preparation Score), cecal intubation rate, use of sedation, number of detected polyps and their histology. Based on these parameters we calculated the polyp detection rate (PDR), ADR, APC, APP.

Endoscopists were not aware of the recorded parameters, but they agreed to participate in this study to find out their level of performance.

### Definition of quality indicators

We used both definitions from ESGE and ASGE guidelines in calculating PDR, ADR, APC and APP to see if there are differences determined by certain indications for colonoscopy, keeping in mind that ASGE reports ADR only for screening colonoscopies, while ESGE reports it for all colonoscopies with a screening or diagnostic indication (Table 1).

**TABLE 1:** ESGE and ASGE definitions of quality indicators.

	ESGE definition Kaminski et al. (2017)	ASGE definition Rizk et al. (2015)
PDR	Number of screening or diagnostic colonoscopies in patients aged 50 years or older in which one or more polyps were detected, divided by the total number of diagnostic colonoscopies	Number of screening colonoscopies in patients aged 50 years or older in which one or more polyps were detected, divided by the total number of screening colonoscopies
ADR	Number of screening or diagnostic colonoscopies in patients aged 50 years or older in which one or more adenomas were detected, divided by the total number of diagnostic colonoscopies	Number of screening colonoscopies in patients aged 50 years or older in which one or more adenomas were detected, divided by the total number of screening colonoscopies
APC	Number of detected adenomas in screening or diagnostic colonoscopies in patients aged 50 years or older divided by the total number of diagnostic colonoscopies	Number of detected adenomas in screening colonoscopies in patients aged 50 years or older divided by the total number of screening colonoscopies
APP	Number of detected adenomas in screening or diagnostic colonoscopies in patients aged 50 years or older divided by the number of diagnostic colonoscopies in which one or more adenomas were detected	Number of detected adenomas in screening colonoscopies in patients aged 50 years or older divided by the number of screening colonoscopies in which one or more adenomas were detected
Exclusion criteria	Colonoscopies with a therapeutic indication or follow-up of inflammatory bowel disease	Patients with family history of CRC or advanced adenoma, or patients with other conditions that classified them as high-risk patients for CRC.

Summarizing these guidelines, we also defined high-quality colonoscopy as an examination complete to cecum in a patient with adequate bowel preparation (Boston Bowel Preparation Score ≥6), performed by a physician with an appropriate ADR, who carefully inspected the colonic mucosa (withdrawal time of at least 6 min) and applied the correct therapeutic procedures.

### Statistical analysis

Statistical analysis was performed using Graph Pad (GraphPad Software, San Diego, CA, United States). Descriptive statistics were reported as mean ± standard deviation (SD), median (interquartile range) and range for continuous variables and as frequency and percentages for discrete variables. The two-tailed Mann-Whitney *U* test was used to compare two continuous variables and the Kruskal–Wallis H test for more than two variables. Violin plots were created to also evaluate the visual differences between two patient groups. Correlations were realized using heatmap (colors range from bright blue for strong positive correlations to bright olive, for strong negative correlations) and Spearman’s correlation coefficient (rho). A *p*-value of less than 0.05 was statistically significant.

## Results

**FIGURE 1:**
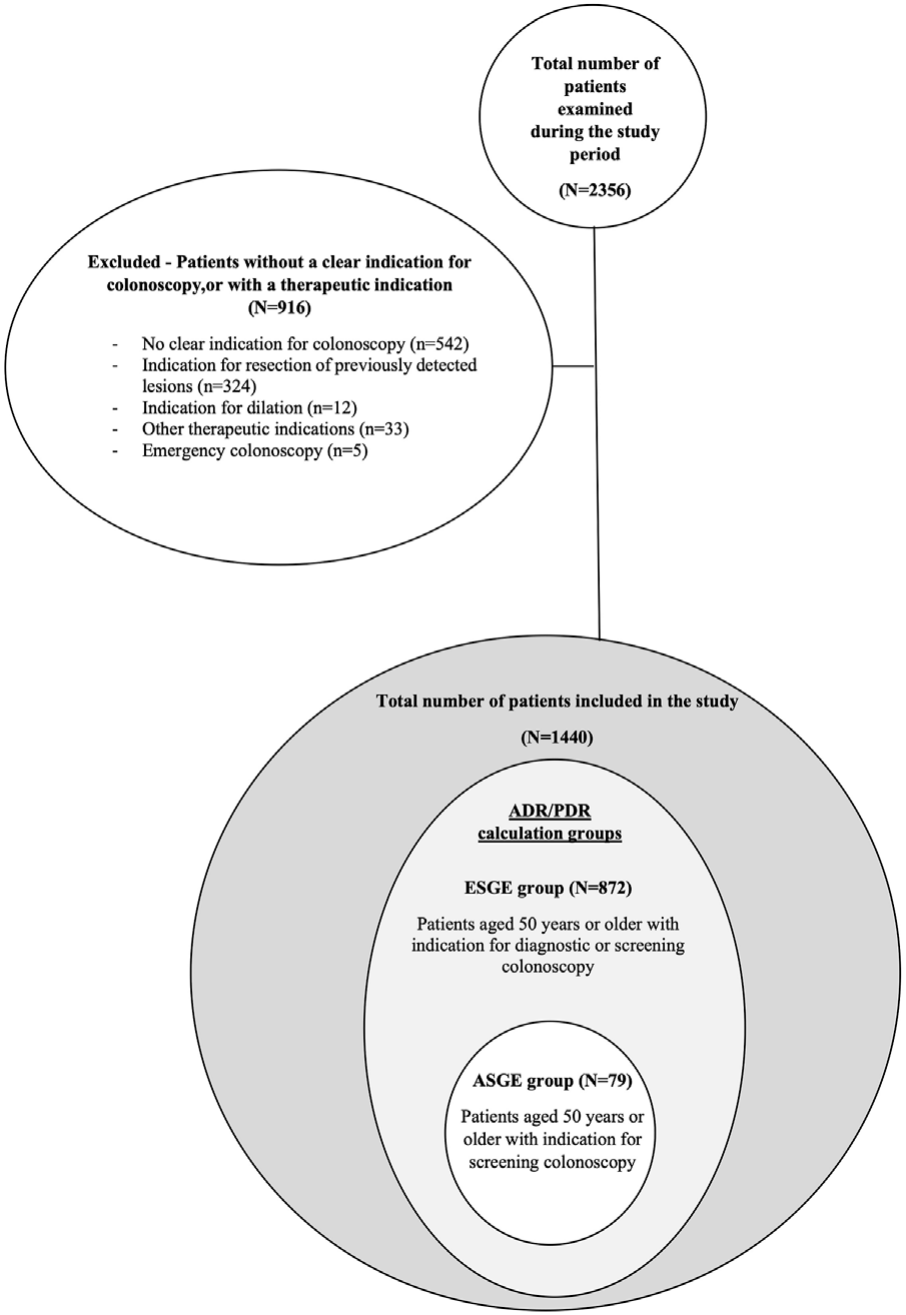
Flow chart of exclusion criteria and ADR/PDR calculations groups.

During the study period a total of 2,356 colonoscopies were performed in the two clinics, 1,440 of which were included in the study. The most frequent exclusion criterion was the lack of an indication for colonoscopy. A flow chart of exclusion criteria and study groups is represented in Figure 1.

A list of the recorded indications for colonoscopy is shown in Table 2. The percentage of screening colonoscopies (all ages) was 14.38%, 6.32% of which were patients with a family history of CRC or advanced adenoma, and 1.46% were patients with previously positive FOBT.

**TABLE 2:** List of recorded indications for colonoscopy.

	Patients	Percentage (%)
Lower gastrointestinal bleeding	281	19.51
Abdominal pain	240	16.67
Post polypectomy follow-up	148	10.28
Diarrhea	121	8.40
Post CRC follow-up	106	7.36
Constipation	97	6.74
Screening (positive family history)	95	6.60
Screening (average risk population)	91	6.32
Other reasons	89	6.18
Proctalgia	53	3.68
Abnormality on imaging study	41	2.85
IBD follow-up	36	2.50
Screening (positive FOBT)	21	1.46
Anemia	21	1.46

Time slot for colonoscopy was 1 h for all examinations, regardless of the indication.

The overall rate of adequate bowel preparation (Boston Bowel Preparation Score ≥6) was 90.34%, with lower scores among patients who used a single dose administration of purgative.

Withdrawal time was recorded in less than half of the diagnostic procedures, so we did not include it in the study.

Characteristics of the endoscopists are shown in Table 3. Only three of them had performed more than 300 examinations during the study period.

**TABLE 3:** Physician characteristics.

	Physician 1	Physician 2	Physician 3	Physician 4	Physician 5
Age, years	47	42	37	57	36
Experience, years	18	14	10	30	9
Number of colonoscopies performed during the study period	243	813	252	381	667
Cecal intubation rate	92.15%	98.20%	96%	98.73%	93.25%
ADR_ESGE_	36.67%	49.46%	37.39%	40.48%	50.47%
PDR_ESGE_	40.00%	52.72%	43.48%	40.48%	62.46%
APC_ESGE_	0.80	1.01	0.59	0.86	1.05
APP_ESGE_	2.18	2.03	1.58	2.12	2.08
ADR_ASGE_	—	70.59%	42.86%	—	49.09%
ADR_ASGE-male_	—	91.67%	25.00%	—	38.46%
ADR_ASGE-female_	—	20.00%	66.67%	—	58.62%
PDR_ASGE_	—	76.47%	42.86%	—	58.18%
APC_ASGE_	—	2.12	0.71	—	0.84
APP_ASGE_	—	3.00	1.67	—	1.70

For the calculation of PDR, ADR, APC and APP 872 patients according to ESGE definition and 79 patients according to ASGE definition were included. Further on we will refer to these patients as the ESGE and ASGE groups.

The ESGE group of patients had a mean age (±SD) of 62.72 (±8.05) years. In this group the overall PDR was 54.02% and ADR was 47.36%. After applying the Kruskal–Wallis H test, no significant differences were noted between physician subgroups regarding age and gender of the patients, only regarding sedation and rate of adequate bowel preparation. One endoscopist did not use sedation, while the others used sedation in over 90% of the procedures, as seen in Table 4.

**TABLE 4:** Characteristics of the patients in ESGE group.

	Physician 1	Physician 2	Physician 3	Physician 4	Physician 5	*p*-value
	*N* = 30	*N* = 368	*N* = 115	*N* = 42	*N* = 317	
Age	62.50 ± 7.83	62.76 ± 7.86	61.58 ± 7.07	66.10 ± 10.65	62.67 ± 8.15	0.183
	61 (55–69.25)	62 (57–68)	61 (56–66)	65 (59–71.50)	62 (56–68)	
	51–77	50–100	50–83	52–110	50–88	
Gender, male	11 (36.7%)	153 (41.6%)	44 (38.3%)	20 (47.6%)	148 (46.7%)	0.411
Sedation						<0.0001
Midazolam	21 (70%)	340 (92.4%)	111 (96.5%)	34 (81%)	—	
Propofol	6 (20%)	11 (3%)	—	6 (14.3%)	—	
No	3 (10%)	17 (4.6%)	4 (3.5%)	2 (4.8%)	317 (100%)	
Sedation, yes	27 (90%)	351 (95.4%)	111 (96.5%)	40 (95.2%)	0 (0%)	
Rate of adequate bowel preparation	94.11%	86.11%	80.50%	97.46%	87.61%	0.002

The ASGE group of patients had a mean age (±SD) of 60.42 (±5.86) years. Regarding age and gender, no differences were observed between patients. Use of sedation was different depending on the evaluated endoscopist, as seen in Table 5.

**TABLE 5:** Characteristics of the patients in ASGE group.

	Physician 2	Physician 3	Physician 5	*p*-value
	*N* = 17	*N* = 7	*N* = 55	
Age	61.18 ± 4.92	57.86 ± 4.53	60.51 ± 6.25	0.366
	61 (56.5–65)	58 (53–60)	61 (56–66)	
	54–70	53–66	50–79	
Gender, male	12 (70.6%)	4 (57.1%)	26 (47.3%)	0.241
Sedation				<0.0001
Midazolam	2 (11.8%)	7 (100.00%)	-	
No	15 (88.2%)	-	317 (100%)	
Sedation, yes	15 (88.2%)	7 (100.00%)	0 (0%)	<0.0001
Rate of adequate bowel preparation	86.59%	85.66%	87.18%	0.002

**FIGURE 2:**
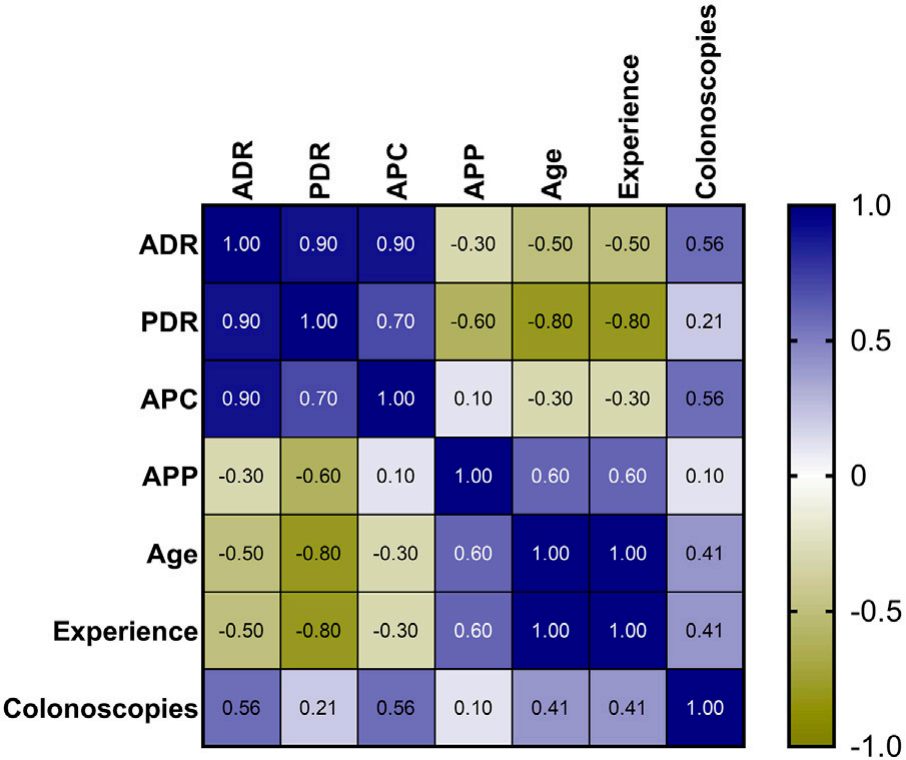
Heatmap of correlations between the indicators of endoscopists.

In the ESGE group there was a strong positive correlation between ADR and PDR (rho = 0.90) and between ADR and APC (rho = 0.90), but without a significant *p*-value due to the small number of endoscopists included in our study (0.083 > 0.05). (Figure 2).

**FIGURE 3:**
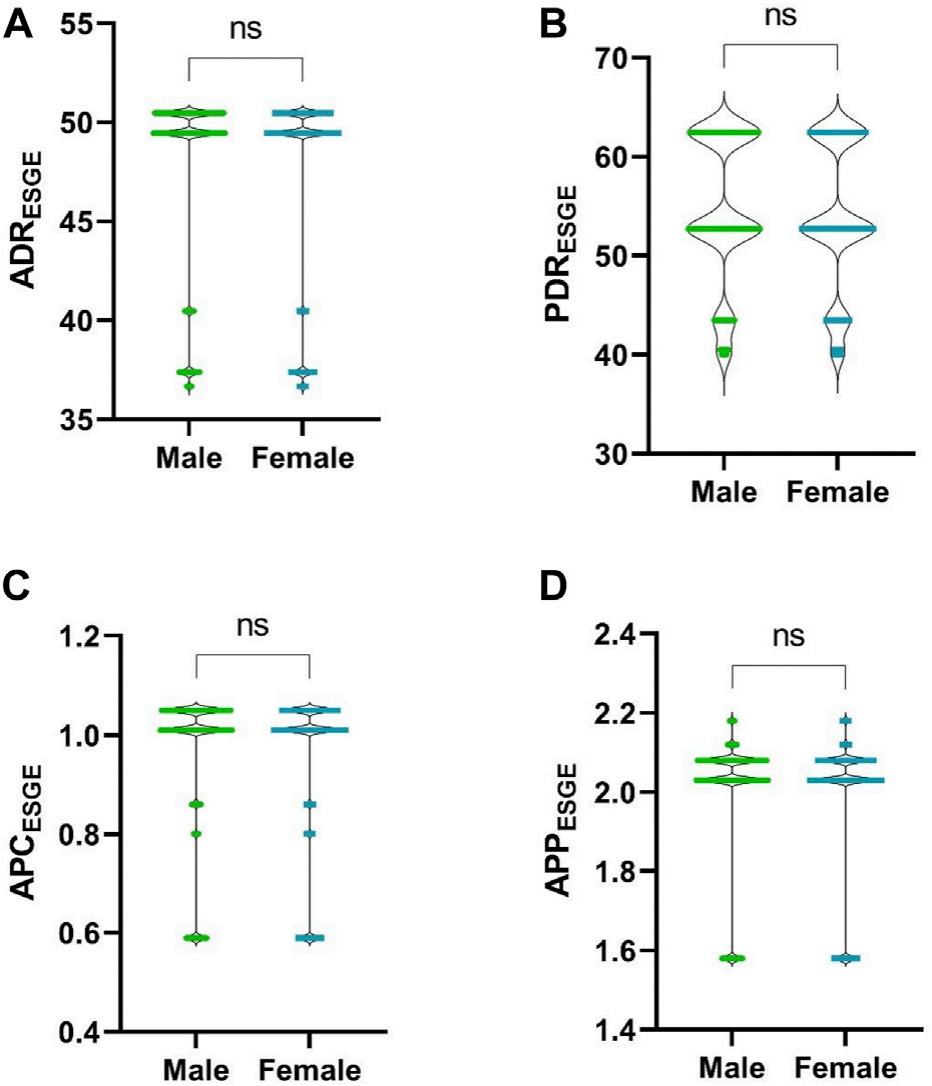
Comparison between sedated and non-sedated patients. **(A)**, ADR; **(B)**, PDR; **(C)**, APC; **(D)**, APP. ****, *p-*value <0.0001.

In the same group we observed significant differences between sedated and non-sedated patients: better ADR (median of sedated vs. non-sedated, 49.46 vs. 50.47), PDR (52.72 vs. 62.46), APC (1.01 vs. 1.05) and APP (2.03 vs. 2.08) were obtained for non-sedated patients (Figure 3). Cecal intubation rate (CIR) was higher when colonoscopies were performed with sedation: OR = 20.62 (95% CI, 6.64–64.18), *p*-value <0.0001.

**FIGURE 4:**
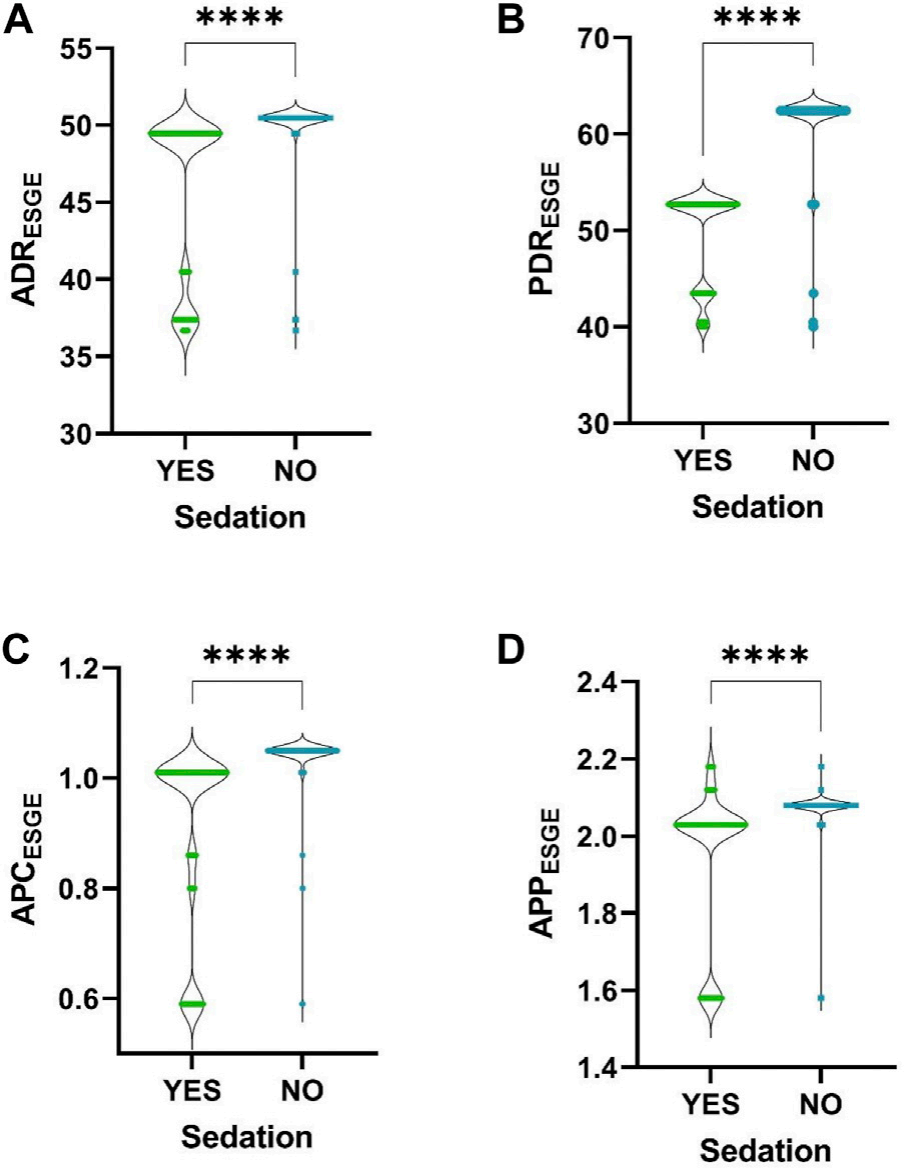
Comparison between male and female patients. **(A)**, ADR; **(B)**, PDR; **(C)**, APC; **(D)**, APP. ns, *p-*value >0.05.

Among the patients in the ESGE group, taking gender into account (376, 43%, male vs. 493, 57%, female), no differences were observed for PDR (*p-*value = 0.1233), ADR (*p-*value = 0.0945), APC (*p-*value = 0.0977) or APP (*p-*value = 0.1364), (Figure 4).

**FIGURE 5:**
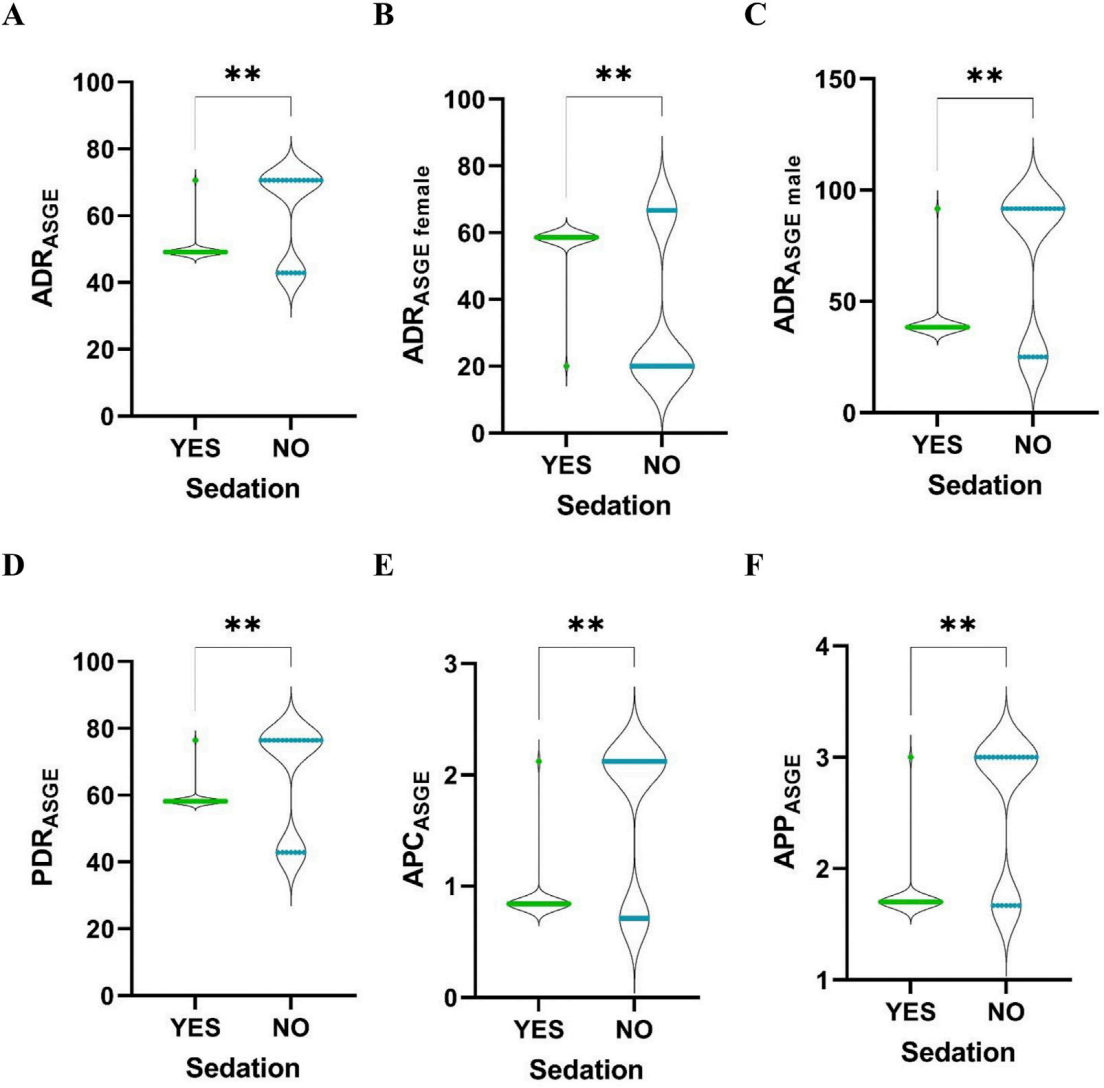
Comparison between sedated and non-sedated patients. **(A)**, ADR; **(B)**, ADR for female; **(C)**, ADR for male; **(D)**, PDR; **(E)**, APC; **(F)**, APP. **, *p-*value <0.01.

In the ASGE group we noted significant differences between sedated and non-sedated patients: a higher ADR (median of sedated vs. non-sedated, 49.09 vs. 70.59), ADR for male patients (38.46 vs. 91.67), PDR (58.18 vs. 76.47), APC (0.84 vs. 2.12) and APP (1.7 vs. 3.0) in the case of non-sedated patients. A higher ADR was noted for female (58.62 vs. 20.00) sedated patients (Figure 5).

In the ESGE group we also wanted to see which of the indications for colonoscopy significantly influence quality indicators. The one with the highest impact on PDR and ADR calculations are diarrhea, screening (especially in case of positive FOBT), and proctalgia. (Table 6).

**TABLE 6:** Correlation between the indication for colonoscopy and PDR, ADR, APC, and APP calculation.

Examination reason	N total = 872 number (percentage)	*p-*value			
		PDR	ADR	APC	APP
Anemia	15 (1.72%)	0.147	0.182	0.151	0.434
Constipation	61 (7.00%)	0.610	0.590	0.627	0.979
Diarrhea	62 (7.11%)	0.004	0.003	0.002	0.013
Abdominal pain	139 (15.94%)	0.303	0.286	0.371	0.664
Lower gastrointestinal bleeding	128 (14.68%)	0.932	0.827	0.894	0.565
Abnormality on imaging study	35 (4.01%)	0.056	0.050	0.050	0.049
Proctalgia	28 (3.21%)	0.056	0.046	0.054	0.167
Screening (positive FOBT)	19 (2.18%)	<0.001	<0.001	<0.001	<0.001
Screening (average risk population)	80 (9.17%)	<0.001	<0.001	<0.001	0.001
Screening (positive family history)	40 (4.59%)	0.023	0.034	0.052	0.532
Post CCR follow-up	92 (10.55%)	0.718	0.737	0.858	0.733
Post polypectomy follow-up	123 (14.11%)	0.291	0.312	0.217	0.216
Other reasons	50 (5.73%)	0.203	0.234	0.354	0.374

We also conducted crude and adjusted linear regression analysis to fully examine the association between ADR and different indications for colonoscopy. As such, ADR was negatively associated with diarrhea and positively associated with the indication for screening in the adjusted analysis. Their coefficients barely changed and, most importantly, kept the direction of the association as in unadjusted analysis (Table 7).

**TABLE 7:** Crude and adjusted linear regression models for ADR calculation.

Examination reason	N total = 872 number (percentage)	Crude coefficients		Adjusted coefficients	
		β (95% CI)	*p-*value	β (95% CI)	*p-*value
Anemia	15 (1.72%)	−1.34 (−3.88,1.21)	0.303	—	-
Constipation	61 (7.00%)	0.48 (−0.82,1.77)	0.472	—	-
Diarrhea	62 (7.11%)	−1.73 (−3.0,−0.44)	0.008	−1.47 (−2.74,−0.19)	0.025
Abdominal pain	139 (15.94%)	−0.05 (−0.95,0.86)	0.920	—	-
Lower gastrointestinal bleeding	128 (14.68%)	0.15 (−0.79,1.08)	0.757	—	-
Abnormality on imaging study	35 (4.01%)	0.43 (−1.25,2.12)	0.613	—	-
Proctalgia	28 (3.21%)	−1.15 (−3.02,0.73)	0.229	—	-
Screening (positive FOBT)	19 (2.18%)	3.12 (0.87,5.38)	0.007	3.19 (0.95, 5.43)	0.005
Screening (average risk population)	80 (9.17%)	1.92 (0.78,3.06)	0.001	1.88 (0.74, 3.02)	0.001
Screening (positive family history)	40 (4.59%)	−0.3 (−1.88,1.28)	0.707	—	-
Post CCR follow-up	92 (10.55%)	−0.12 (−1.19,0.96)	0.834	—	-
Post polypectomy follow-up	123 (14.11%)	−0.54 (−1.49,0.4)	0.261	—	-
Other reasons	50 (5.73%)	−0.66 (−2.08,0.76)	0.361	—	-

CI, confidence intervals.

## Discussions

The demographic data of the patients included in the study were very similar between all five endoscopists, both in terms of average age and gender, with a higher percentage of women examined.

The characteristics of the physicians were different both in terms of age and years of experience, but also in terms of the average number of colonoscopic examinations per year. We did not identify a correlation between the ADR value and any physician characteristics, although in previous similar studies they represented an important variable in the calculation of ADR (Lee et al., 2014; Jover et al., 2016; James et al., 2018). A slightly higher ADR was observed in younger doctors, with a higher average number of colonoscopies per year.

The most common indications for colonoscopy were lower digestive bleeding and abdominal pain. The percentage of screening examinations was low compared with other similar studies (Gupta et al., 2010; Boroff et al., 2017), the number of screening colonoscopies for patients with average risk of CRC representing only 6.32% of the total examinations included in the study. In the mentioned studies, the percentage of screening colonoscopies varied between 8.4% and 49.2%. For this reason, the calculation of ADR according to ASGE definition generated results with few statistically significant data. To be able to correctly calculate the ADR according to the ASGE definition, a follow-up for at least 1 year of each endoscopist under the given conditions would be necessary, or it would be necessary to introduce automatic digital calculation of the ADR. The only statistically significant conclusion from the ASGE patient group was that the ADR is higher among examinations performed without sedation than those performed with sedation. The same could be observed in the ESGE group. In the literature, there are conflicting data regarding the influence of sedation on ADR. In some studies ADR was significantly higher when colonoscopies were performed with sedation (Khan et al., 2020; Zhang et al., 2020), and in others ADR was not influenced by sedation (Bannert et al., 2012; Lee et al., 2014; Zhao et al., 2020) but the frequency of major complications increased among sedated patients (Zhao et al., 2020). We have not found studies in which sedation negatively influences ADR, and the data we obtained may represent a particularity that deserves to be studied further or may be a false positive finding determined by the limitations of the study. Cecal intubation rate was higher when colonoscopies were performed with sedation which is in accordance with the data from the literature. There is a new measure of quality in colonoscopy that is being studied, called Performance Indicator of Colonic Intubation (PICI), which is defined as the rate of cecal intubation without significant discomfort and use of minimal sedation (Nass et al., 2021). The study of this indicator shows that there is an interest in reducing the amount of sedation during colonoscopy, or in performing as many examinations as possible without sedation, while maintaining the comfort of the patient and the quality of the examination. Our data may be of interest for the further study of PICI.

The ESGE group of patients was the reference group in this study. In this group overall PDR was 54.02% and ADR was 47.36%, significantly higher than the minimum values recommended by ESGE (Kaminski et al., 2017). The rate of adequate bowel preparation was lower than the minimum recommended standard by ESGE in three out of five examiners and we would have expected it to generate lower PDR and ADR values, but we did not find a positive correlation between these indicators. We tried to find an explanation for the increased ADR and PDR values and thus performed an analysis of the indicators related to the indication for colonoscopy. We found that ADR was negatively associated with diarrhea and positively associated with screening. None of the other indications for colonoscopy had a significant influence on its calculation. This means that the calculation method proposed by ESGE does not increase the ADR or PDR values, compared to the method proposed by ASGE. On the contrary, in our patient group, these indicators were higher when reporting was done only at screening colonoscopies. The negative influence of diarrhea on ADR may be due to the overuse of colonoscopy in cases of infectious or functional diarrhea.

Time slot for colonoscopy was higher than the minimum standard recommended by ESGE (30 min for clinical and primary screening colonoscopy; 45 min for colonoscopy following positive FOBD) (Kaminski et al., 2017), and perhaps this led to a more thorough examination of the colonic mucosa, with more frequent detection of polyps and a higher ADR.

There were no differences in the calculation of ADR, PDR, APC and APP regarding patient gender, so we consider that it is not necessary to establish a minimum target of these indicators according to gender, contrary to ASGE recommendations (Rizk et al., 2015).

### Limitations of the study

Considering that this was an observational study, it was not possible to impose the parameters that had to be followed and thus indicators such as withdrawal time or indication for colonoscopy were poorly recorded. This led to the exclusion of many patients from the study.

The total number of examinations as well as the number of endoscopists was low and some of the results had no statistical significance. Nevertheless, the power test was done and assuming an alpha level of 0.05, the correlations between the endoscopists indicators yielded the power between 84% and 95% for the different analysis.

The study followed the activity of endoscopists over a period and not several of consecutive examinations. Thus, significant differences appeared between physicians in terms of the number of examinations performed, which led to results without statistical significance in some instances. We believe that for a correct ADR calculation it is necessary to include at least 300 consecutive diagnostic colonoscopies for each physician, and in our study only three endoscopists met this condition.

## Conclusion

Even if in Romania the quality in colonoscopy is not routinely monitored, according to our data, endoscopists seem to exceed the minimum standards recommended by international societies. The lower rate of screening colonoscopies does not influence the ADR calculation and any of the definitions proposed by international societies can be used for its assessment.

Also, ADR correlated well with PDR, APC and APP and we think that it could be used as the only quality indicator in countries where there is no quality monitoring in endoscopy until other indicators can be evaluated and calculated automatically.

## Data Availability

The original contributions presented in the study are included in the article/Supplementary Material, further inquiries can be directed to the corresponding author.
